# The Boundary Between Volume and Surface-Driven Magnetic Properties in Spinel Iron Oxide Nanoparticles

**DOI:** 10.1186/s11671-022-03737-w

**Published:** 2022-10-11

**Authors:** Giuseppe Muscas, Francesco Congiu, Giorgio Concas, Carla Cannas, Valentina Mameli, Nader Yaacoub, Rodaina Sayed Hassan, Dino Fiorani, Sawssen Slimani, Davide Peddis

**Affiliations:** 1 Department of Physics, University of Cagliari, Cittadella Universitaria Di Monserrato, S.P. 8 Km 0.700, 09042 Monserrato, CA Italy; 2 Università Degli Studi Di Cagliari, Dipartimento Di Scienze Chimiche E Geologiche, and INSTM, Cittadella Universitaria Di Monserrato, S.P. 8 Km 0.700, 09042 Monserrato, CA Italy; 3 IMMM, Le Mans Université, CNRS UMR-6283, Avenue Olivier Messiaen, 72085 Le Mans, France; 4 Department of Physics, Faculty of Science, Lebanese University, Beirut, Lebanon; 5 Dipartimento Di Chimica E Chimica Industriale, Università Degli Studi Di Genova, Via Dodecaneso 31, 1-16146 Genoa, Italy; 6 Istituto Di Struttura Della Materia-CNR, 00015 Monterotondo Scalo, RM Italy

**Keywords:** Nanomagnetism, Nanoparticles, Ferrites, Surface properties, Exchange bias

## Abstract

Despite modern preparation techniques offer the opportunity to tailor the composition, size, and shape of magnetic nanoparticles, understanding and hence controlling the magnetic properties of such entities remains a challenging task, due to the complex interplay between the volume-related properties and the phenomena occurring at the particle’s surface. The present work investigates spinel iron oxide nanoparticles as a model system to quantitatively analyze the crossover between the bulk and the surface-dominated magnetic regimes. The magnetic properties of ensembles of nanoparticles with an average size in the range of 5–13 nm are compared. The role of surface anisotropy and the effect of oleic acid, one of the most common and versatile organic coatings, are discussed. The structural and morphological properties are investigated by X-ray diffraction and transmission electron microscopy. The size dependence of the surface contribution to the effective particle anisotropy and the magnetic structure are analyzed by magnetization measurements and in-field Mössbauer spectrometry. The structural data combined with magnetometry and Mössbauer spectrometry analysis are used to shed light on this complex scenario revealing a crossover between volume and surface-driven properties in the range of 5–7 nm.

**Graphical Abstract:**

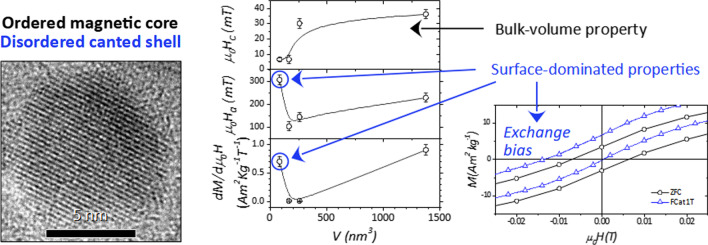

**Supplementary Information:**

The online version contains supplementary material available at 10.1186/s11671-022-03737-w.

## Introduction

On entering the nanometer-scale regime, the magnetic properties of condensed matter show substantial differences with respect to the bulk state, leading to “new physics” [1–3] and applications [4, 5]. In magnetism, several phenomena are related to the nanoscale, such as the dimensions of magnetic domains and the length of exchange coupling interactions. For this reason, since a few decades ago, nanostructured magnetic materials are the object of great attention. Among them, nanoparticles (NPs) are unique complex physical objects: In these systems, a multidomain organization is energetically unfavorable and single-magnetic-domain particles are formed [6]. Within such domains, all atomic spins act coherently as a single magnetic “supermoment,” a gigantic replica of individual atomic spins [7]. The static and dynamic magnetic properties of monodomain NPs are mainly governed by the magnetic anisotropy energy. This quantity represents the energy barrier that the magnetic supermoment needs to overcome to freely rotate in space. In the simplest case of a spherical particle with uniaxial anisotropy, the magnetic anisotropy energy depends on the angle θ between the applied field and the anisotropy easy axis according to:

1 $$\Delta E = K_{{{\text{eff}}}} V\sin^{2} \theta$$

The maximum value Δ*E* = *K*_eff_ *V* depends on the effective anisotropy constant *K*_eff_ and the particle volume V. At a certain temperature, the magnetic supermoment undergoes a thermally activated transition with a characteristic relaxation time *τ* [2, 8]:

2 $$\tau = \tau_{0} \exp \left[ {\frac{{K_{{{\text{eff}}}} V}}{{k_{B} T}}} \right]$$

For a given experimental technique, one can identify the temperature below which the system appears as “static,” i.e., when the relaxation time *τ* is equal to the experimental measuring time *τ*_*m*_. This identifies the so-called blocking temperature *T*_*B*_:

3 $$T_{B} = \frac{{K_{{{\text{eff}}}} V}}{{k_{B} \ln \left( {\tau_{m} /\tau_{0} } \right)}}$$

Therefore, *T*_*B*_ depends on the measuring time and is proportional to both the anisotropy constant and the particle volume. Above this temperature, the relaxation time becomes shorter than the measuring one, the particle moment thermally fluctuates, and the observed magnetization results in an average value over the measuring time. This behavior is analogous to paramagnetism, but with different time and magnetization scales, and, for this reason, it is called “superparamagnetism” (SPM) [7, 9]. In addition, interparticle interactions can eventually induce a collective behavior among supermoments according to a disordered spin-glass (SG)-like [10] or ordered ferromagnetic (FM)-like model [11].

Single-domain magnetic nanoparticles are not only a platform to study magnetism at the nanoscale, but they also offer peculiar properties at the base of a multitude of technological fields, such as nanomedicine [12] (e.g., magnetic sensors [13], bio-imaging [14], drug delivery [15], therapeutic hyperthermia [16, 17]), ferrofluid technology [18], catalysts [19], color imaging [20], high-density magneto recording [21], and, recently, they have found an important role in thermoelectric systems [22, 23]. Moreover, a renewed interest in nanoparticle-based magnetic systems is driven by their potential use for 3D magnonic metamaterials [24, 25], in particular exploiting the self-assembling of interacting magnetic nanoparticles [26, 27]. In this framework, understanding the physics of magnetic nanoparticles and controlling their magnetic properties represent hot topics of large technological importance.

For bulk materials, the effective anisotropy is mainly determined by the magnitude and symmetry of the crystallographic anisotropy (magneto-crystalline anisotropy) [28]. According to Eq. 1, the effective anisotropy energy has a direct dependence on the average particle volume [29]. However, at the nanoscale, an additional level of complexity emerges at the particles’ surface [30]. Here, the breaking of the lattice symmetry induces a reduction in the local atomic coordination number, due to missing atoms, leading to a significant modification of the bulk magnetic order, which is very sensitive to any variation of distance/angle among atomic moments. Hence, this translates into distinct surface properties such as lower saturation magnetization and higher anisotropy [31–33]. In addition, oxidation phenomena can extend from the surface to the core, gradually changing the structural and magnetic properties [34]. The emergence of the surface anisotropy is strictly connected to the breaking of the crystal lattice and hence to the shape of the nanoparticles [35]. Interestingly, the recent work of Mamiya et al. [36] has compared spherical, cubic, and octahedral particles, with sizes above 7 nm, observing that the shape anisotropy still plays a minor role compared to the magnetocrystalline one. Anyway, upon reducing particle size to a few nanometers, the surface anisotropy contribution can overcome the bulk magneto-crystalline one, significantly increasing the overall effective anisotropy value [6] and widening its distribution [37]. Such surface effects are intrinsic to the nanoscale, and they are present even in particles with high crystal quality [38]. On the other hand, surface effects can be exploited even in larger magnetic nanostructures controlling the growth of thin surface layers [39].

The surface properties of magnetic NPs can be tuned by employing a coating of organic molecules. By selecting the proper ligand, surfactant agents can improve the atomic coordination on the surface, repairing the missing bonds, with significant effects on the magnetic properties, such as a controlled decrease of anisotropy and an increase of saturation magnetization [31, 40, 41]. The organic coating can also be effective in protecting particles from oxidation, in dispersing them in a specific solvent, in avoiding aggregation in ferrofluids, and in functionalizing them for specific purposes (e.g., in biomedicine through biocompatible ligands) [41].

Despite the numerous observations evidencing the strong influence of surface phenomena and the effect of organic coatings, the use of magnetic nanoparticles in technological applications requires a more quantitative approach to investigate the emergence of surface-dominated regimes, and to quantify the effect of organic surfactants on the surface magnetic disorder. To shed light on this complex scenario, in the present work, we investigate the interplay between the volume and the surface magnetic properties on four ensembles of spinel iron oxide particles. These samples serve as a model system for small magnetic nanoparticles. In addition, spinel iron oxides, offering chemical and thermal stability, as well as rich crystal chemistry for fine-tuning the magnetic properties, represent a versatile material of large technological relevance [42–45]. The samples have been prepared by high-temperature thermal decomposition (HTD) of acetylacetonate precursors, a synthesis method that provides a high level of control over the structural and physical properties of magnetic NPs [46, 47]. The average particles’ size of the samples has been tuned in the range of ≈ 5–13 nm, where the variation of the surface/volume ratio leads to the emergence of significant surface/interface effects. In order to draw the boundary between the bulk volume-dominated and the surface-dominated regime, we have carried out a combined analysis of the magnetic properties investigated by SQUID magnetometry and of the magnetic structure by Mössbauer spectrometry with a large applied magnetic field. In addition, to study the effect of organic coating, highlighting its effect on the surface magnetic disorder, three of the samples (MAG1, MAG2, MAG3) have been synthesized using an oleic acid (OA) coating, while one (MAG4) has been prepared without OA.

## Experimental

### Synthesis

To prepare 5-nm spinel iron oxide nanoparticles, iron(III) acetylacetonate (Janssen Chimica 99%, 2 mmol), 1,2-hexadecanediol (Sigma-Aldrich 97%, 10 mmol), oleic acid (Sigma-Aldrich 90%, 6 mmol), oleylamine (Sigma-Aldrich < 70%, 6 mmol), and phenyl ether (Sigma-Aldrich 99%, 20 ml) were mixed into a three-neck, round-bottom flask and magnetically stirred. The mixture was heated gradually to 200 °C and kept at this temperature for 30 min. Then, the temperature was increased rapidly up to 300 °C, and the mixture was kept at this temperature for 30 min under reflux. The starting solution changed color from orange-red to dark black, suggesting the formation of magnetite nanoparticles. The mixture was cooled to room temperature by removing the heat source. Ethanol (40 ml) was then added to destabilize the mixture, and the black product was separated via centrifugation. After several washing cycles with ethanol, the powder was finally dispersed in hexane. Before magnetic measurements, the dispersion was destabilized once again with ethanol, recovered by centrifugation, and dried at 40 °C overnight to evaporate residual alcohol.

With the same method, 7-nm spinel iron oxide nanoparticles (MAG2) were prepared but using benzyl ether (20 ml) instead of phenyl ether. For this sample, the reflux time was 2 h at 200 °C and 1 h at 300 °C.

For 8-nm spinel iron oxide nanoparticles (MAG3), iron(III) acetylacetonate (2 mmol) was added to a hexane solution containing 84 mg of MAG2 particles that acted as seeds for the growth of larger particles. The synthesis procedure was the same as MAG2.

For 13-nm spinel iron oxide nanoparticles (MAG4), iron(III) acetylacetonate (3 mmol) and oleylamine (45 mmol) were mixed in benzyl ether (15 ml) and kept under reflux for 1 h at 110 °C, and for 1 h at 300 °C.

### Measurements and Data Treatment

X-ray diffraction (XRD) analysis was carried out using a Seifert diffractometer with a θ-θ Bragg–Brentano geometry, with Cu-K_α_ wavelength. The samples, in form of powder, were analyzed on a zero-background silicon holder in the 2θ range of 25—70°.

For transmission electron microscopy (TEM) analysis, the samples’ powders were dispersed in octane and submitted to an ultrasonic bath. Then, the suspensions were dropped on carbon-coated copper grids and observed with a JEOL 200CX microscope, operating at 200 kV. High-resolution (HR) TEM images were obtained with a JEM 2010 UHR microscope equipped with a Gatan Imaging Filter (GIF) and a 794 slow-scan CCD camera. The recorded images were analyzed with the software ImageJ [48]. The contours of more than 200 particles were manually defined for each sample, and, thanks to the automated measurement suite of the software, the exact particle's projected area was measured. Then, assuming a spherical particle shape and knowing the area value, the diameter D was calculated for each particle. A particle size distribution was calculated, with a bin size of 1 nm, compatible with the Sturges’ rule [49], except for MAG4, where the rule application shows its limitation and results in over smoothing the distribution [50]. Finally, a log-normal function was fitted to the size distribution:

4 $$P = \frac{A}{{D w \sqrt {2\pi } }}\exp - \left[ {\frac{{\ln^{2} \left( {{\raise0.7ex\hbox{$D$} \!\mathord{\left/ {\vphantom {D {D_{{{\text{TEM}}}} }}}\right.\kern-\nulldelimiterspace} \!\lower0.7ex\hbox{${D_{{{\text{TEM}}}} }$}}} \right)}}{{2w^{2} }}} \right]$$

where *A* is the area of the peak, w, the standard deviation of the natural logarithm of the variable D, and <D_TEM_> is the median of the log-normal distribution, which gives an estimation of the average particle size. To estimate the broadening of the particles’ size distribution of the samples, the coefficient of variation (COV) was calculated [51]. The latter represents the ratio between the standard deviation and the mean of particle size, which, for a log-normal distribution, corresponds to:

5 $${\text{COV}} = \sqrt {e^{{w^{2} }} - 1}$$

The exact fraction of the magnetic phase (i.e., free of surfactant) was determined by thermogravimetric analysis and simultaneous differential thermal analysis (TGA-SDTA) measurements performed using a Mettler-Toledo TGA/SDTA 851. The data were collected in the range of 25–1000 °C with a heating rate of 10 °C min^−1^ under oxygen flow (flow rate of 50 ml/min).

^57^Fe Mössbauer spectra were recorded using a ^57^Co/Rh γ-ray source mounted on an electromagnetic transducer with velocity modulated according to a triangular waveform. The samples consist of a thin layer of powder pressed inside a sample holder. The spectra were obtained at 10 K in an 8 T field oriented parallel to the γ–beam. The data were analyzed by using the program Mosfit. The hyperfine structure was modeled by means of a least-square fitting procedure involving Zeeman sextets composed of Lorentzian lines. To describe the broadening of lines, several magnetic subcomponents were considered. Isomer shift, quadrupolar shift, line width, and effective field values were free during the refinement as well as the intensities of intermediate lines (2,5) resulting from the angle between the hyperfine field and the γ-beam. The ratio of the absorption areas of external/internal lines was systematically fixed to 3. The isomer shift (IS) values were referred to that of α-Fe at 300 K.

DC magnetization measurements were performed by a Quantum Design MPMS 5 and a PPMS DynaCool magnetometers. For the magnetization vs temperature analysis, the zero field cooled (ZFC) and field cooled (FC) procedures were followed. To perform ZFC measurements, the sample is first cooled from room temperature to 5 K in zero field; then, the magnetization (M_ZFC_) is recorded warming up from 5 to 300 K, with a static applied magnetic field of 2.5 mT. With the same magnetic field applied, the M_FC_ was recorded during the subsequent cooling from 300 to 5 K.

M versus H curves were measured in the interval ± 5 T of applied field at the temperature of 5 K. The saturation magnetization *M*_*S*_ was extrapolated fitting the law of approach to saturation to the curves at high field [52]:

6 $$M = M_{S} \left( {1 - \frac{A}{H} - \frac{B}{{H^{2} }}} \right)$$

where *A* and *B* are constant parameters. The field dependence of the remanent magnetization was measured using the IRM (isothermal remanent magnetization) and DCD (direct current demagnetization) protocols. According to the IRM protocol, the sample, in a demagnetized state, was cooled in a zero magnetic field down to 5 K. A small magnetic field was applied for 10 s and, after switching it off, the corresponding remanent magnetization was recorded. The process was repeated using increasing field steps up to + 5 T. In a DCD measurement, the sample, cooled at 5 K, is first saturated in a − 5 T field applied for 10 s. Then, the remanence was measured similarly to the IRM protocol, but increasing the field from − 5 T to + 5 T.

## Results and Discussion

**Fig. 1:**
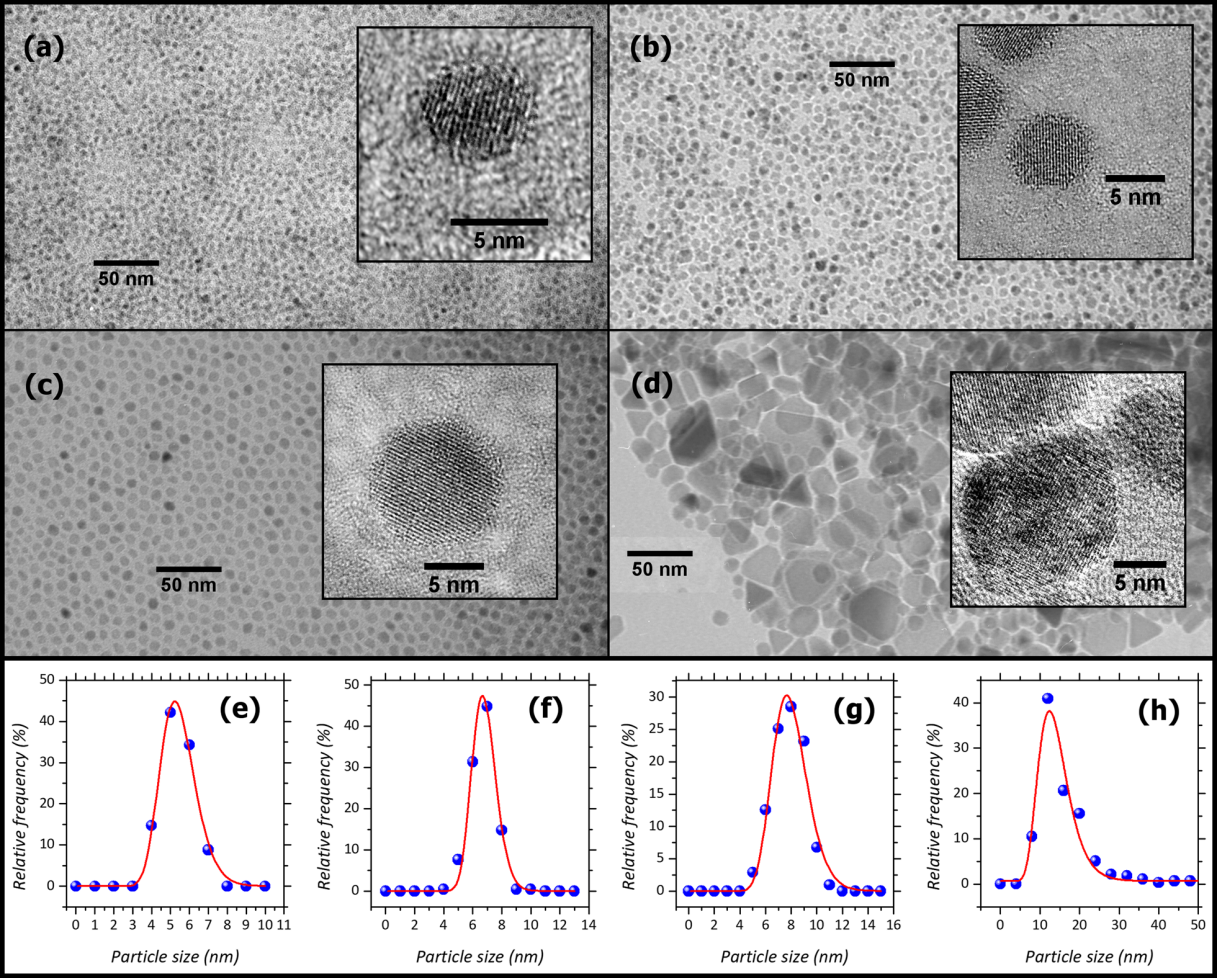
TEM images of MAG1 (**a**), MAG2 (**b**), MAG3 (**c**), and MAG4 (**d**), with inset high-resolution images of single particles representative of the morphology and crystallinity of the samples. The corresponding particles’ size distributions are reported in panels **e**, **f**, **g**, and **h** for MAG1, MAG2, MAG3, and MAG4, respectively. The frequency counts of the measured size are represented as blue spheres, while the continuous red line describes the log-normal fit to the data

All samples exhibit a crystalline structure characteristic of spinel iron oxide (Additional file 1: Fig. S1). However, the XRD pattern alone cannot discriminate between magnetite (Fe_3_O_4_, PDF card 19–0629) and maghemite (γ-Fe_2_O_3_, PDF card 25–1402). Despite the two crystalline structures should present different lattice parameters (γ-Fe_2_O_3_ ~ 0.833 nm, Fe_3_O_4_ ~ 0.840 nm), our samples show intermediate values ranging from about 0.835 to 0.840 nm without any visible trend among them. The presence of distortion at the nanoscale, non-ideal cationic distribution, and vacancies can significantly affect this value, preventing identifying unambiguously the two crystalline phases. Mössbauer spectrometry (reported later) will confirm the maghemite nature of the samples. The high crystallinity of particles is evident from the HR-TEM images (Fig. 1); it is worth noting that sample MAG3, prepared by a seed-mediated growth process, does not exhibit defects in the crystalline structure due to the shell growth. This will be confirmed later also by the magnetic characterization, very sensitive even to small internal structural inhomogeneities and/or strain effects [53]. Samples MAG1, MAG2, and MAG3 show almost spherical particles and narrow size distribution (Fig. 1a–c), while MAG4 owns particles with more irregular shapes (Fig. 1d) and broader size distribution as evidenced by the large value of the coefficient of variation (Table 1). This highlights that the mixture of oleic acid-oleylamine-hexadecanediol used to prepare the first three samples is effective in tuning the morphology of particles.

**Table 1:** Average particles diameter <D_TEM_> , polydispersity evaluated as the coefficient of variation (COV), the temperature corresponding to the maximum of the ZFC curve (*T*_max_), and the irreversible temperature between ZFC and FC curves (*T*_irr_) are reported

Sample	<*D*_TEM_> (nm)	COV (%)	*T*_max_ (K)	*T*_irr_ (K)
MAG1	5.4(1)	0.17(1)	17(1)	100(20)
MAG2	6.8(1)	0.12(1)	20(1)	200(30)
MAG3	7.9(1)	0.17(2)	53(2)	200(20)
MAG4	13.5(3)	0.35(4)	–	–

For MAG4 these two temperatures are above 300 K, the maximum value employed for the measurements

### Evolution of Magnetic Anisotropy

**Fig. 2:**
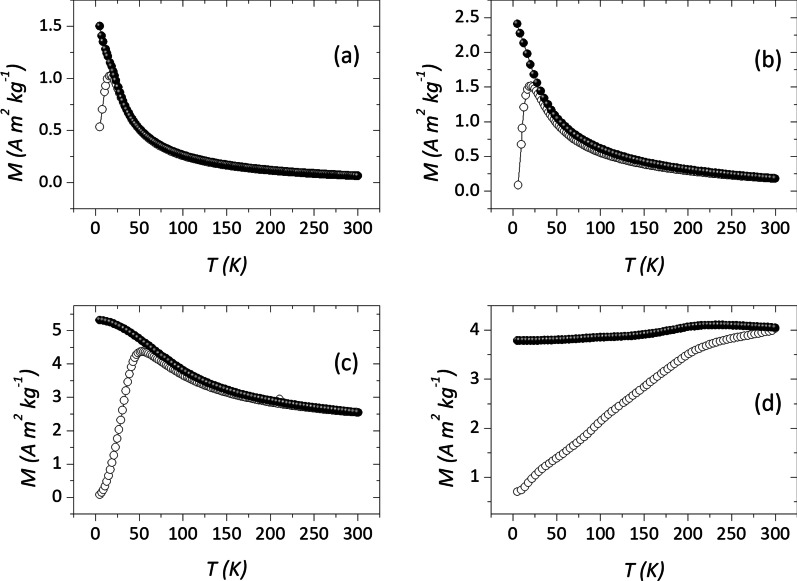
ZFC (empty circles) and FC (full circles) curves for samples MAG1 (**a**), MAG2 (**b**), MAG3 (**c**), and MAG4 (**d**), measured with an applied field of 2.5 mT

The evolution of the zero field cooled (ZFC) and field cooled (FC) curves reported in Fig. 2 offers a first overview of the dependence of the anisotropy energy on the average particle volume. For an ensemble of non-interacting particles with equal size, the temperature corresponding to the maximum of the ZFC curve (*T*_*max*_) is equivalent to the blocking temperature (T_B_), which is proportional to the particles’ volume. However, the unavoidable presence of a volume distribution shifts the peak position to a higher temperature [1]. The irreversibility temperature (*T*_irr_, taken as the temperature at which the difference between FC and ZFC becomes smaller than 3%) is related to the blocking of the particles with the highest anisotropy (the biggest particles, if the magnetocrystalline anisotropy is dominant) [54]. As expected, both *T*_max_ and *T*_irr_ show a general increment with increasing particles’ size (Table 1), and for MAG4, they are above room temperature, due to the large particles’ size. The FC magnetization curve shows a rapid increase below *T*_max_ (according to a Curie-like behavior) for MAG1 and MAG2, a slower increase (slope decreasing with decreasing temperature) for MAG3, and a very weak temperature dependence range for MAG4 without showing a maximum in ZFC magnetization. This suggests a change from non-interacting or weakly interacting particles’ behavior (for MAG1 and MAG2) to that of moderately interacting particles (for MAG3), to collective magnetic behavior, where interparticle interactions are dominant (for MAG4). Interparticle interactions provide an additional contribution to the effective magnetic anisotropy and then to the *T*_max_ and *T*_*B*_ values [55].

**Fig. 3:**
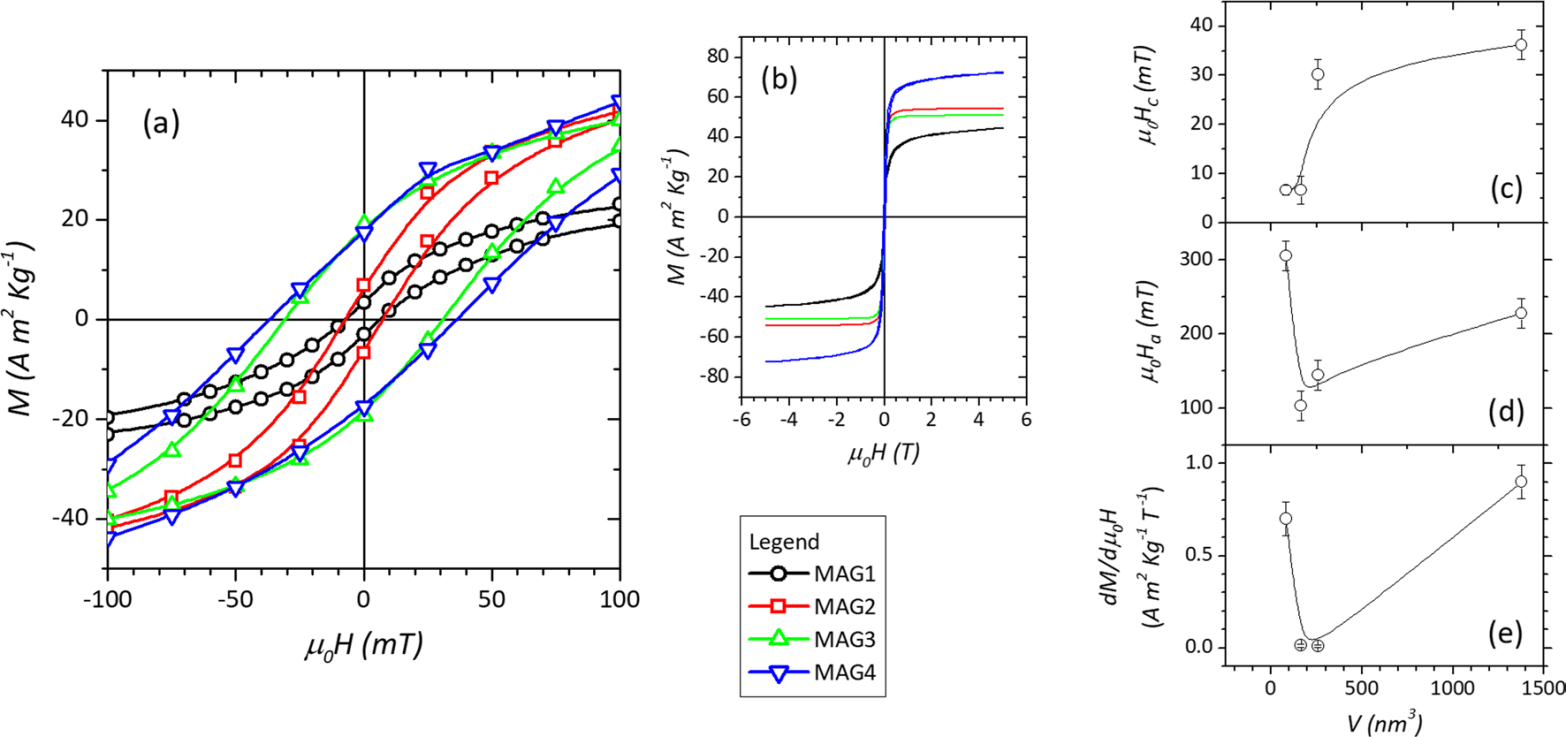
**a** A magnification at low field of the M versus H curves measured at 5 K for all samples with **b** the full range of measurement. The size dependence of coercive field (*µ*_0_*H*_*C*_), anisotropy field (µ_0_*H*_a_), and the high-field susceptibility (dM/dµ_0_H) measured at 5 T are reported in panels **c**, **d**, and **e**, respectively, with lines used as a guide to the eye

The magnetization versus field M(H) curves measured at 5 K (Fig. 3a, b and data in Table 2) show hysteretic behavior due to the blocked state of the particles’ moment. From the curves, both the coercive field *H*_C_ and the anisotropy field *H*_a_ are estimated. The coercive field is related to the effective average anisotropy energy of the particles. The anisotropy field (*H*_a_) is the field corresponding to the merging of the up and down branches of the magnetization curves, here estimated as the field at which their difference is below 1% of their maximum value.

**Table 2:** Saturation magnetization (*M*_*S*_), coercive field (*µ*_0_*H*_*C*_), anisotropy field (µ_0_*H*_a_), and magnetic susceptibility (dM/dµ_0_H) at 5 T

Sample	*M*_*S*_ (A m^2^ kg^−1^)	*µ*_0_*H*_*C*_ (mT)	*µ*_0_*H*_a_ (mT)	dM/dµ_0_*H* at 5 T (A m^2^ kg^−1^ T^−1^)
MAG1	48(2)	7(1)	310(70)	0.70(9)
MAG2	54(2)	7(3)	100(60)	0.014(9)
MAG3	51(2)	30(6)	140(90)	0.011(7)
MAG4	75(2)	36(6)	230(90)	0.90(9)

The coercive field increases with increasing the particle size with a non-monotonous trend. On the other hand, the anisotropy field first sharply decreases from MAG1 to MAG2 and then increases with increasing size. The observed values, as well as those of T_max_ and T_irr_, indicate that the volume of the particles plays an important role on the effective magnetic anisotropy [56]. However, since H_C_ and H_a_ do not follow a linear trend with respect to the particle volume, as it would be expected from Eq. 1, this suggests the presence of an additional contribution to the anisotropy, the relative role changing with the particle size.

For a better understanding of the evolution of anisotropy with the particle size, the volume dependence of the high-field susceptibility dM/dµ_0_H, measured at 5 T, has been analyzed (Fig. 3e). Its value, proportional to the anisotropy of the canted surface spins [57], reproduces quite well the anisotropy field trend. It strongly decreases from MAG1 to MAG2 and remains stable for MAG3, indicating a much more important role of surface anisotropy in the smallest particles. MAG4 exhibits the largest value, potentially connected to an extended magnetic surface disorder. The OA used for the synthesis of the first three samples, due to its nature as a π-acceptor [58, 59], increases the crystal field splitting energy, thus reducing the spin-orbit coupling and so the local surface anisotropy [41, 60]. This can explain the low values of high-field susceptibility recorded for MAG2 and MAG3 samples coated by OA, and the large value of MAG4, being this synthesized and eventually coated only with oleylamine, which owns a donor nature with an opposite effect with respect to OA [58, 60], Besides, the irregular multifaced morphology of MAG4 particles could further enhance the surface contribution by introducing additional shape anisotropy. On the other hand, such anisotropy should not be determinant since MAG1, coated by OA and consisting of regular spherical particles, shows the second largest value of dM/dµ_0_H. This can be the signature of the crossover from a magnetic regime dominated by the volume contribution, to a new one controlled by the surface phenomena.

### Interparticle Interactions

For samples MAG1, MAG2, and MAG3, the presence of the OA coating sets an average interparticle distance that prevents any direct contact and thus exchange interparticle interactions [61]. On the other hand, direct contact and exchange interaction are not excluded for the particles of MAG4, partially coated by oleylamine (Additional file 1: Sect. S1). In an ensemble of randomly distributed nanoparticles with average magnetic moment *μ*_*NP*_ and average separation *d*, the energy due to dipole–dipole interactions can be approximated to [62]:

7 $$E_{{{\text{dip}}}} \approx \frac{{\mu_{0} }}{4\pi }\frac{{\mu_{{{\text{NP}}}}^{2} }}{{d^{3} }}$$

**Fig. 4:**
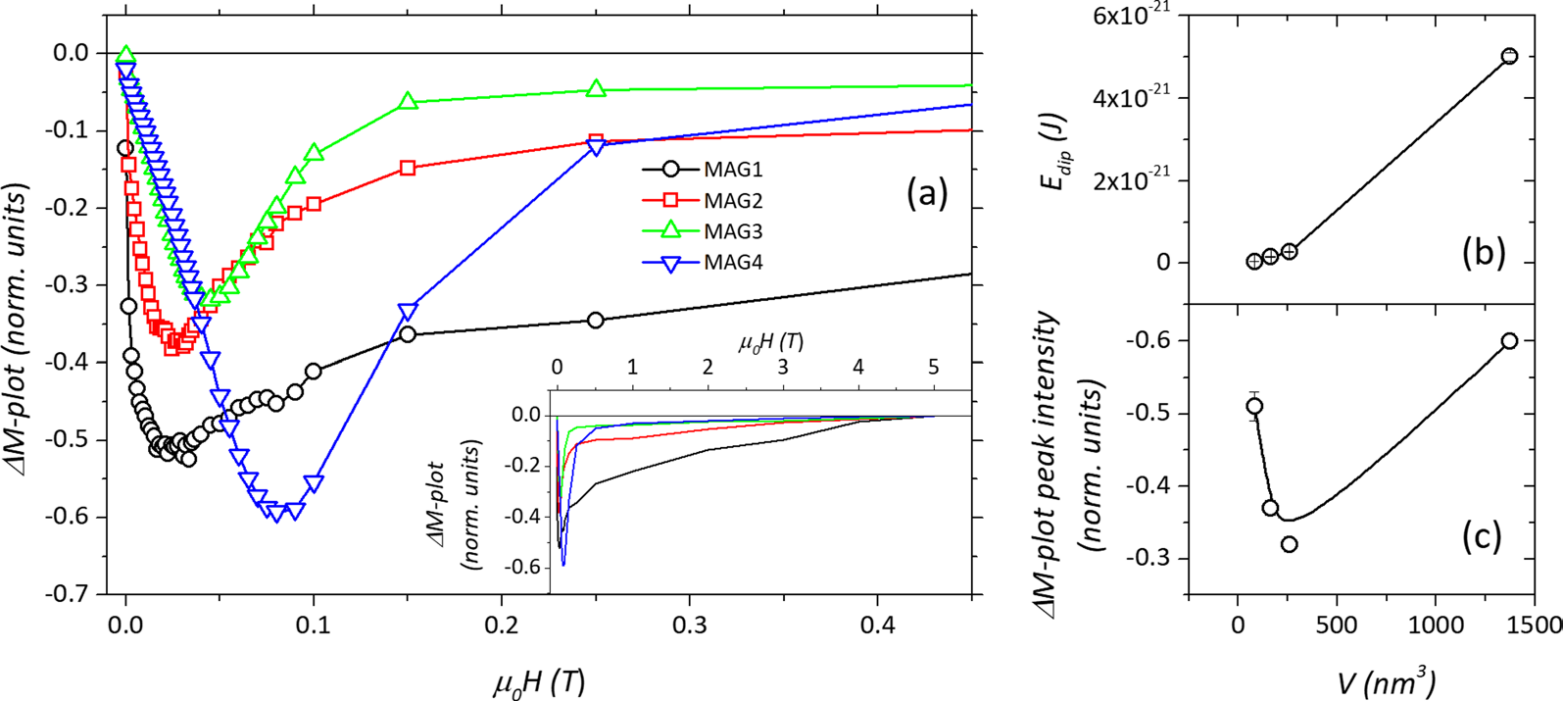
**a** Low-field details of the ΔM-plots at 5 K. The full range of the measurements is reported in the inset. **b** The dipolar interaction energy and **c** the ΔM-plots peaks’ intensity is plotted as a function of the average particle’s volume, with lines used as a guide to the eye

The mean value of dipolar energy *E*_dip_ is calculated using Eq. 7 assuming a point dipole model, i.e., considering *d* as particles’ center-to-center distance, including the thickness of the OA coating of about 2 nm [59], and defining the magnetic moment of a single-domain particle as *μ*_NP_ = *M*_*S*_* V*. The results are reported as a function of volume in Fig. 4. The IRM and DCD curves provide an additional picture of the interaction regime (Additional file 1: Fig. S3). For non-interacting single-domain particles with uniaxial anisotropy and magnetization reversal by coherent rotation, the two remanence curves are related via the Wohlfarth equation [63]. To explicitly reveal deviations from a non-interacting case, Kelly et al. [64] proposed the equation in the form:

8 $$\Delta M = m_{{{\text{DCD}}}} \left( H \right) - 1 + 2m_{{{\text{IRM}}}} \left( H \right)$$

where *m*_DCD_(H) and *m*_IRM_(H) represent the reduced terms *M*_DCD_(*H*)/*M*_DCD(5 T)_ and *M*_IRM_(*H*)/*M*_IRM(5 T)_, with M_DCD(5 T)_ and M_IRM(5 T)_ being the remanence values for a reversal field of 5 T for the DCD and IRM curves, respectively.

All the investigated samples show a strong negative deviation (Fig. 4a), due to demagnetizing effects resulting from magnetic dipolar interparticle interaction [65]. It is interesting that even for MAG4, showing the largest negative Δ*M* deviation, dipolar interactions clearly dominate even on possible exchange interactions. The amplitude of the negative peaks in the graph, proportional to the effect of the magnitude of the interaction on the reversal field [46], shows an interesting anomaly. While the *E*_dip_ predicted for the samples exhibits a monotonous trend as a function of particle volume (Fig. 4b), the intensity of the Δ*M* plots (Fig. 4c) shows an unexpected high interacting regime for MAG1. Additional negative contributions to the Δ*M* plots can arise from strong magnetocrystalline anisotropy with cubic symmetry [66]. However, nanoscale finite-size effects usually suppress such symmetry, as we can observe in our samples. Indeed, the *M*(*H*) shows a ratio between remanence and saturation magnetization well-below the theoretical cubic anisotropy value of 0.83 [67]. A more likely explanation for such an anomaly is the presence of a surface shell with canted spins with distinct magnetic anisotropy. Recently, a similar experimental observation, confirmed also by Monte Carlo simulations, has been explained with the coupling between a magnetically disordered surface shell and an ordered core, responsible for an additional negative contribution to ΔM plots [65]. A similar effect has been also observed in bi-magnetic exchange-coupled systems [68]. Noteworthy, the comparison of interparticle interactions in the set of samples under investigation requires special care, due to the different average magnetic anisotropy in each sample. Indeed, the experimental observations are the results of the combined effect of the interparticle interaction energy and the effective single particle anisotropy energy and their specific ratio [69, 70].

### Magnetic Structure and Magnetic Anisotropy

**Fig. 5:**
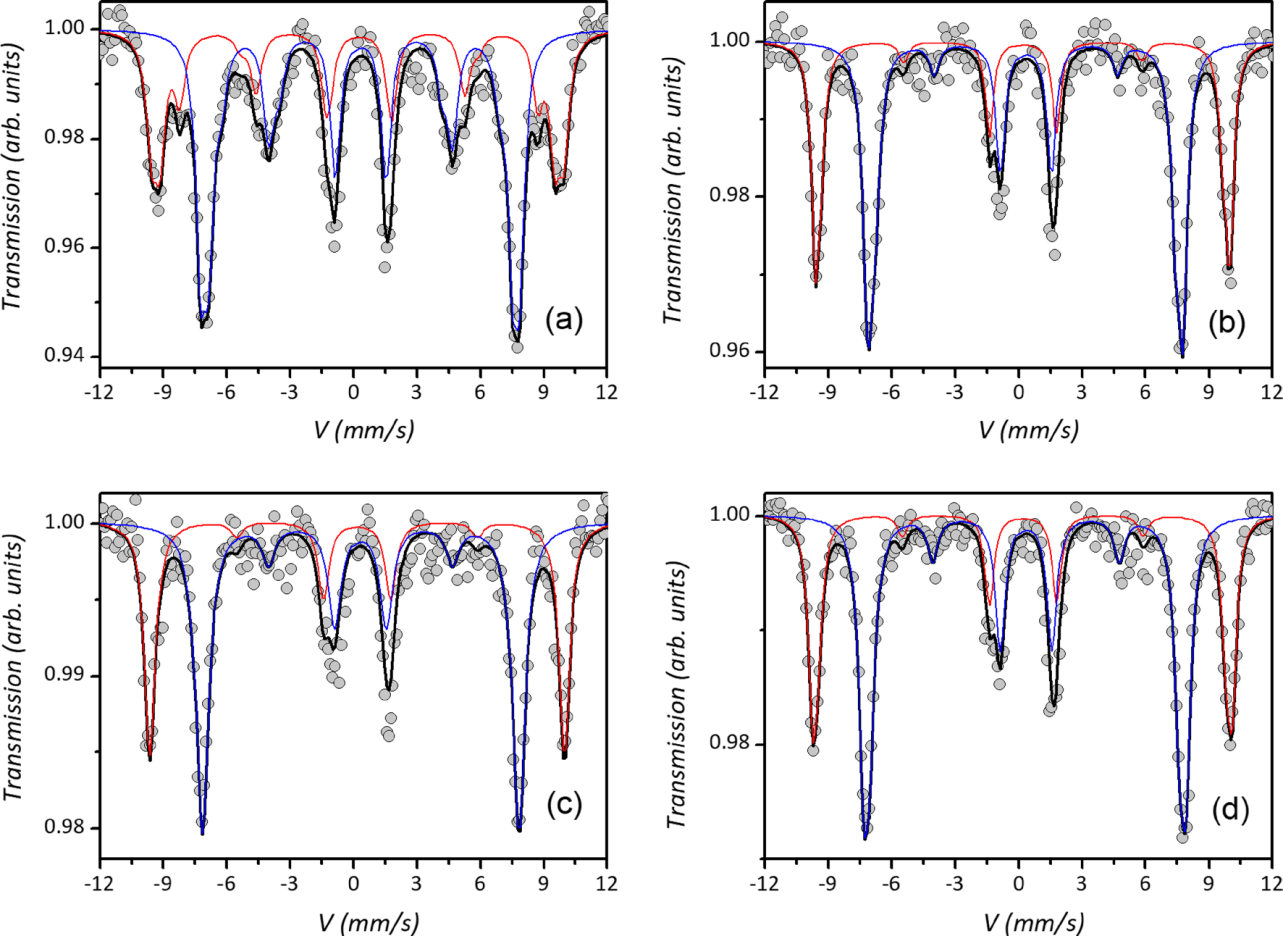
Mössbauer spectra measured at 10 K under an applied field of 8 T for samples MAG1 (**a**), MAG2 (**b**), MAG3 (**c**), and MAG4 (**d**). A sum of two sextets, one for the tetrahedral (red line) and one for the octahedral (blue line) component of the spectra, have been fitted to the experimental data (circles). A black line describes the total fit to the experimental data (grey circles)

High-field Mössbauer spectrometry measurements [71, 72] provide detailed information on the magnetic structure also in nanostructured systems [73]. The spectra were recorded at 10 K applying a magnetic field of 8 T parallel to the γ-ray direction (Fig. 5). Under these conditions, the temperature is low enough to suppress the thermally activated magnetization switching and the spectra show a magnetic hyperfine component indicating that the particle moments are in the blocked state. Using a high applied magnetic field during the measurements, the fitting to the experimental data allows distinguishing two sextets associated with Fe in tetrahedral *T*_*d*_ and octahedral *O*_*h*_ sites of the spinel structure [74, 75]. The applied field is usually added to the *T*_*d*_ site hyperfine field and subtracted from the *O*_*h*_ site, being negative the dominant Fermi contact term. Since the magnetic field is applied parallel to the γ-ray direction, the nonzero intensity of the second and fifth lines of the sextet provides evidence of a canted magnetic structure [75, 76] (see Additional file 1: Sect. 3, for additional details).

From the modeling of the in-field Mössbauer spectra, a direct estimation of the hyperfine parameters (i.e., hyperfine field, isomer shift, quadrupole shift), effective field *B*_eff_, and the spin canting angle (*θ*_cant_, angle defined by the direction of the effective field and the γ-beam direction) have been obtained for both *T*_d_ and *O*_h_ iron components. Hyperfine field and isomer shift values are typical of maghemite nanoparticles for all the samples (i.e., all iron is oxidized to Fe^3+^, and vacancies are distributed in both *T*_d_ and *O*_h_ sites in place of Fe^2+^) [77].

**Fig. 6:**
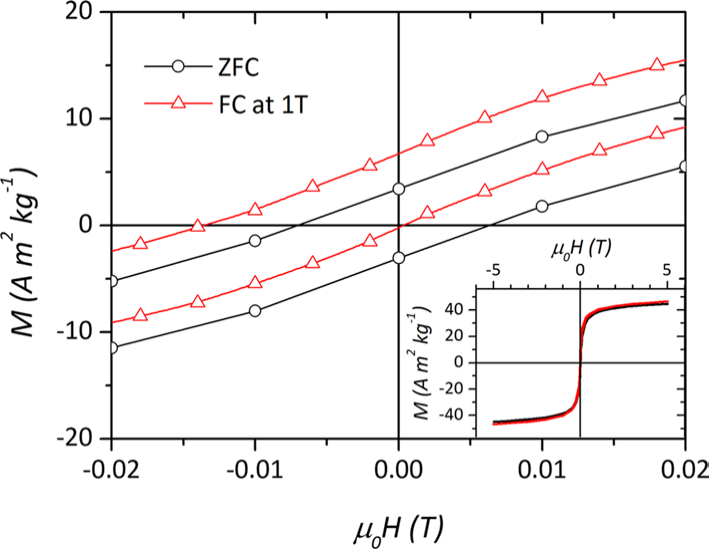
Low-field magnification of *M*(*H*) loops recorded at 5 K after cooling the sample MAG1 from 300 K in zero field (black line and circles) and in 1 T (red line and triangles). In the inset, the extended field range of measurement

All samples display spin canting even if with a different magnitude: MAG2 and MAG3 present the lowest values of canting, MAG4 shows a significantly larger value, but MAG1 shows the largest mean canting angle (Table 3). This would indicate that in so small particles (≈ 5 nm), the surface-disordered structure extends to a large fraction of the particle volume so that the magnetic behavior is actually dominated by the surface-induced effects, as evidenced by its large value of anisotropy field. Further confirmation of this crossover to a surface-dominated regime comes from another interesting phenomenon. The M(H) curve recorded at 5 K after cooling the sample in a field of 1 T shows exchange bias, e.g., a negative horizontal shift of − 6.2(8) mT (Fig. 6). Such behavior is due to the exchange coupling between a disordered frozen surface and the interior core of the particles [65]. Interestingly, Levy et al. [53] noted an exchange bias effect in 8 nm particles prepared by seed-mediated growth similarly to MAG3. It was justified by the presence of internal strain linked to inhomogeneous shell growth occurring at multiple nucleation sites. To verify this phenomenon on MAG3, we have recorded *M*(*H*) curve after field cooling such as for MAG1. However, no sign of bias has been observed (Additional file 1: Fig. S5), possibly due to the higher final temperature used for the growth of our sample (300 vs. 250 °C), which induced a more coherent growth of the shell resulting in a homogeneous structure.

**Table 3:** From the fitting of Mössbauer spectra, the % area of each component, their mean individual isomer shift (*δ*), mean hyperfine field (*B*_hf_), and the average canting angle (*θ*_cant_) are evaluated

Sample	Site	%	*δ* (mm s^−1^)	B_hf_ (T)	*θ*_cant_ (°) ± 10°
MAG1	*T*_*d*_	39	0.41	50.5	38
	*O*_*h*_	61	0.49	51.2	
MAG2	*T*_*d*_	39	0.36	52.6	19
	*O*_*h*_	61	0.49	52.9	
MAG3	*T*_*d*_	38	0.32	52.9	22
	*O*_*h*_	62	0.48	53.6	
MAG4	*T*_*d*_	38	0.33	53.0	28
	*O*_*h*_	62	0.49	53.2	

For more details about hyperfine parameters, like mean individual quadrupolar 2*ε* shift and mean canting angle, see Additional file 1: Table S1 in SI

To explain the complex magnetic structure of maghemite emerging from Mössbauer spectrometry, Coey [76] proposed the nanoparticle model of a magnetically ordered core surrounded by a disordered/canted shell. This model has been extensively applied to interpret experimental data [29, 30, 33, 78–80] and confirmed by simulations [81, 82]. In particular, recent works by Krycka et al. [30] and Negi et al. [79] probed, by different experimental techniques, the presence of a 1-nm-thick disordered shell on spinel iron oxide nanoparticles. Assuming perfect collinear spins in the core and canted spins localized only on the particles’ surface, we can estimate the thickness *t* of such canted shell from the particle diameter and the average canting angle extracted from Mössbauer data [75] (detailed description in Additional file 1: Sect. 3.1):

9 $$t = \left[ {1 - \left( {1 - \frac{3}{2}\sin^{2} \vartheta } \right)^{{{\raise0.7ex\hbox{$1$} \!\mathord{\left/ {\vphantom {1 3}}\right.\kern-\nulldelimiterspace} \!\lower0.7ex\hbox{$3$}}}} } \right]\frac{D}{2}$$

MAG4, due to the absence of OA, has the thickest disordered shell, ≈ 0.8 nm. MAG2 and MAG3 display the smallest values of *t*, ≈ 0.2, and ≈ 0.3 nm, respectively, in agreement with their small average canting angle and high-field susceptibility. As observed by Salafranca et al. [33], OA restores a large part of the lost local coordination at the surface. Interestingly, our analysis allows us to quantify that the thickness of the disordered shell is reduced by more than half. Finally, despite the OA coating, MAG1 owns a value of *t* ≈ 0.7 nm. It is worth mentioning that the estimated thickness values translate into a canted spin structure involving around 20% of the total particle volume for MAG2 and MAG3, 30% for MAG4, and 60% for MAG1. This can explain why the latter shows an overall magnetic behavior strongly dominated by the surface canted structure. Indeed, its relative extension is large enough to represent a distinct magnetic phase coupled with the ordered magnetic core, resulting in an exchange bias effect.

The value of coercivity provides an estimation of the strength of the magnetic field needed to reverse the bulk of the magnetization. In this sense, the increase in particle volume is the dominating factor, which determines the increment in the values. On the other hand, the anisotropy field represents the extra resistance to overcome to partially realign the canted spins, as recently observed by Zákutná et al. [80] This explains why MAG1 exhibits the lowest coercive field, due to the smallest particle volume among the samples, but also the largest value of anisotropy field, caused by the large fraction of total volume represented by the canted surface. This demonstrates that by reducing the particle’s size from 7 nm of MAG2 to 5 nm of MAG1, the boundary between the volume and surface-dominated regimes has been overcome, and the magnetic behavior of such particles is effectively dominated by the finite-size effects. Interestingly, this observation agrees pretty well with the investigation of Bakuzis et al. [83] on MnFe_2_O_4_, where the surface magnetic anisotropy emerged strongly for particle size below 6 nm, and also with the work of Mamiya et al. [36] on Fe_3_O_4_, where the surface anisotropy plays a minor role for size above 7 nm. A tentative explanation for the limited effect of OA on MAG1 can be proposed based on the reduced radius of curvature of such particles. This can potentially enhance the surface crystalline distortions, and, at the same time, it can limit the regular layer of oleic acid attached to the surface and hence its capability of reordering the surface distortions.

## Conclusions

We have investigated ensembles of maghemite nanoparticles with an average size in the range of 5–13 nm correlating the analysis of their magnetic properties with their specific structure.

Following the conventional Stoner–Wohlfarth model, the anisotropy energy governs the reversal of non-interacting particles, and this energy increases linearly with the particle volume. However, the investigated samples do not follow this trend. Indeed, observing the coercive and anisotropy field values as a function of volume, additional anisotropy terms emerge. The surface anisotropy becomes more influential with decreasing size, becoming dominant for MAG1, the sample with the smallest particles. Interparticle and intraparticle (i.e., exchange coupling between magnetically ordered core and disordered shell) interactions also contribute to the effective anisotropy, increasing with increasing the particle size, even more for MAG4 due to the lack of oleic acid coating. Mössbauer spectroscopy under an intense magnetic field allows clarifying the magnetic structure of the samples. All the samples exhibit a surface canted structure, which, due to its intrinsic high anisotropy, partially aligns only under high applied fields, as evidenced by the anisotropy field and high-field susceptibility values. In this picture, the oleic acid coating plays an important role. MAG4 (13.5 nm particles) is the only sample without oleic acid coating and exhibits the thickest magnetic disordered shell, which accounts for ≈ 30% of the total particle volume. Its effect is clearly observed in the high values of anisotropy field and high-field susceptibility. Reducing particle size to 7.9 and 6.8 nm (MAG3 and MAG2, respectively), the relative disordered volume should increase. However, the OA coating on these samples replaces the missing bonds of surface cations, partially restoring their bulk coordination. The thickness of their disordered magnetic shell is then minimized to ≈ 0.3 and 0.2 nm, respectively. Therefore, the anisotropy field and high-field susceptibility are greatly reduced. Below about 6 nm, the OA capability of restoring the local structure appears reduced, due to the higher degree of disorder. Indeed, despite the same OA coating, the 5.4-nm particles of MAG1 exhibit a disordered shell of ≈ 0.7 nm. This represents ≈ 60% of the total particle volume. Such a large fraction is not only responsible for the largest anisotropy field among the samples, but also represents a second “hard” magnetic phase that pins the spins of the “soft” core, inducing the exchange bias phenomenon.

This work evidences a crossover between volume and surface-driven properties of magnetic nanostructures, identifying it in the range of 7–5 nm for spinel iron oxide nanoparticles. Our investigation precisely quantifies the degree and extension of the surface magnetic disorder and analyzes the effect of the commonly employed oleic acid coating. Moreover, our results are not limited to maghemite nanoparticles, but the methodology and the general conclusions apply to all nanoparticle-based magnetic systems. This is enormously important since understanding and quantifying the role of the surface/interface of ultra-small systems is crucial for any technological application.

## Supplementary Information

**Additional file 1**: Supporting information including: XRD patterns, TGA-SDTA data and analysis, IRM and DCD data and method, details on Mössbauer spectrometry in high magnetic field, sample MAG3 ZFC and FC hysteresis curves at 5 K.

## Data Availability

The datasets used and/or analyzed during the current study are available from the corresponding author on reasonable request.

## Abbreviations

NPs: Nanoparticles
SPM: Superparamagnetism
SG: Spin-glass
FM: Ferromagnetic
HTD: High-temperature thermal decomposition
OA: Oleic acid
XRD: X-ray diffraction
TEM: Transmission electron microscopy
HR: High resolution
COV: Coefficient of variation
TGA-SDTA: Thermogravimetric analysis and simultaneous differential thermal analysis
IS: Isomer shift
ZFC: Zero field cooled
FC: Field cooled
IRM: Isothermal remanent magnetization
DCD: Direct current demagnetization
