# Supplementary material for: The Boundary Between Volume and Surface-Driven Magnetic Properties in Spinel Iron Oxide Nanoparticles
Source: Nanoscale Res Lett. 2022 Oct 11;17:98. doi: 10.1186/s11671-022-03737-w (PMC9554062; doi:10.1186/s11671-022-03737-w)
Supplement: Supplementary file 1 — Additional file 1: Supporting information including: XRD patterns, TGA-SDTA data and analysis, IRM and DCD data and method, details on Mössbauer spectrometry in high magnetic field, sample MAG3 ZFC and FC hysteresis curves at 5 K. [file 11671_2022_3737_MOESM1_ESM.docx]

**The boundary between volume and surface-driven magnetic properties in spinel iron oxide nanoparticles**

Giuseppe Muscas^1^*, Francesco Congiu^1^, Giorgio Concas^1^, Carla Cannas,^2^ Valentina Mameli^2^, Nader Yaacoub^3^, Rodaina Sayed Hassan^3,4^, Dino Fiorani^6^, Sawssen Slimani^5,6^, and Davide Peddis^5,6#^.

^1^Department of Physics, University of Cagliari, Cittadella Universitaria di Monserrato, S.P. 8 Km 0.700, I-09042 Monserrato (CA) Italy

^2^Università degli Studi di Cagliari, Dipartimento di Scienze Chimiche e Geologiche, and INSTM, Cittadella Universitaria di Monserrato, S.P. 8 Km 0.700, I-09042 Monserrato (CA) Italy

^3^IMMM, Le Mans Université, CNRS UMR-6283, Avenue Olivier Messiaen, Le Mans, 72085, France

^4^Lebanese University, Faculty of Science, Department of Physics, Beirut, Lebanon

^5^Università degli Studi di Genova, Dipartimento di Chimica e Chimica Industriale, Via Dodecaneso 31, 1-16146 Genova, Italy

^6^Istituto di Struttura della Materia-CNR, 00015 Monterotondo Scalo (RM), Italy

Corresponding authors:

[*giuseppe.muscas@dsf.unica.it](mailto:*giuseppe.muscas@dsf.unica.it)

[^#^davide.peddis@unige.it](mailto:#davide.peddis@unige.it)

*Supplementary Information*

# Structural characterization





Figure S1. XRD patterns of the iron oxides samples prepared by the high-temperature thermal decomposition process. All samples exhibit a crystalline structure compatible with spinel iron oxide. Nevertheless, the XRD pattern alone cannot discriminate between magnetite (Fe_3_O_4_ , PDF card 19-0629) and maghemite (γ-Fe_2_O_3_ , PDF card 25-1402).

## Thermogravimetric analysis

Thermogravimetric analysis (TGA) gives additional information about the surfactant amount (**Fig. S2(a)**). At low temperature, all samples showed a very small weight loss centered around 100°C and a larger one in the range of 200-500 °C. The first weight loss is coupled with an endothermic peak in the corresponding simultaneous differential thermal analysis (S-DTA) curves (**Fig. S2(b)**), which is related to the evaporation of residual ethanol and water. For the first 3 samples, a series of exothermic peaks are visible in the range of 200-500 °C, related to the distinct events of evaporation and decomposition of a loose external organic layer and a more stable internal one, directly bonded to the particles’ surface [1]. These signals correspond to a weight loss of about 40%, matching roughly to a coating of two layers of oleic acid. MAG2 and MAG3 show sharp exothermic peaks up to 500 °C. Only after the evaporation/decomposition of the first external layer at around 360 °C (boiling point of the free surfactants), a second inner layer is decomposed at a higher temperature with a sharp exothermic peak. On the other hand, the organic surfactant in sample MAG1 is completely decomposed before reaching 400 °C. This suggests a weaker bonding for MAG1, compatible with the reduced reordering effect of the surface magnetic structure observed for this sample. MAG4, the only one prepared without oleic acid, shows a complete decomposition of a small amount (about 8%) of organic surfactant before 400 °C, suggesting a partial coating layer with weak bonding between oleylamine and particles surface compared to oleic acid.





Figure S2. TGA (a) and simultaneous S-DTA (b) for MAG1, MAG2, MAG3, and MAG4 measured in the temperature range of 25-800 °C.

# DCD - IRM curves and ΔM-plots

The field dependence of the remanent magnetization was measured using the IRM (Isothermal Remanent Magnetization) and DCD (Direct Current Demagnetization) protocols. According to the IRM protocol, the samples, in the demagnetized state, were cooled in a zero magnetic field down to 5 K. At this temperature, a small external field was applied for 10 s, then switched off, and, finally, the remanence (*M_IRM_*) was measured. The process was repeated, increasing the field in steps up to 5 T. In a DCD measurement, the initial state was the magnetically saturated one. After cooling the sample at 5 K, an external field of -5 T was applied for 10 s, then it is turned off and the remanence (*M_DCD_*) was measured. As in IRM, a small external field in the opposite direction to magnetization was applied for 10 s and then switched off. Finally, the remanent magnetization was measured. This was repeated increasing the field up to +5 T.





Figure S3. IRM (solid black line) and DCD curves (dashed red line) measured at 5 K for MAG1 (a), MAG2 (b), MAG3 (c), and MAG4 (d).

The analysis of the remanent magnetization curves measured by *IRM* and *DCD* protocols (**Fig. S3**) allows us to investigate the interaction regime among particles. For an assembly of non-interacting single-domain particles with uniaxial anisotropy and magnetization reversal by coherent rotation, the two remanence curves are related via the Wohlfarth equation [2]:

$$m_{DCD}\left( H \right)=1-2m_{IRM}(H) (1)$$

where *m_DCD_(H)* and *m_IRM_(H*) represent the reduced terms *M*_DCD_*(H)/M_DCD(5T)_* and *M*_IRM_*(H)/M_IRM(5T)_*, and *M_DCD(5T)_ and M_IRM(5T)_* are the remanence values for the *DCD* and *IRM* curves for a reverse field of 5 T, respectively. Kelly et al. [3] rewrote the Wohlfarth relation to explicitly reveal deviations from a non-interacting case:

$${\Delta M=m}_{DCD}\left( H \right)-1+2m_{IRM}(H) (2)$$

Negative *ΔM* are usually taken as indicative of the prevalence of demagnetizing (e.g., dipole-dipole) interactions; positive values are attributed to interactions promoting the magnetized state (e.g., direct exchange interactions). Qualitatively, the intensity of such deviation (*I_ΔM_*) can be considered proportional to the strength of the interparticle interactions [4].

# Average canting angle evaluated by means of high field Mössbauer spectrometry

^57^Fe Mössbauer spectra have been recorded at 10 K under a magnetic field of 8 T (*B_ext_*) applied parallel to the γ –beam. When the second and fifth lines have a non-zero intensity, they evidence a canted structure for iron magnetic moments with respect to the applied field (non-collinear magnetic structure) [5, 6]. In the case of a non-collinear spin structure, the measured effective nucleus field (*B_eff_*) differs from the external field (*B_ext_*) due to the average canting angle *ϑ* as graphically illustrated in **Fig. S4**.


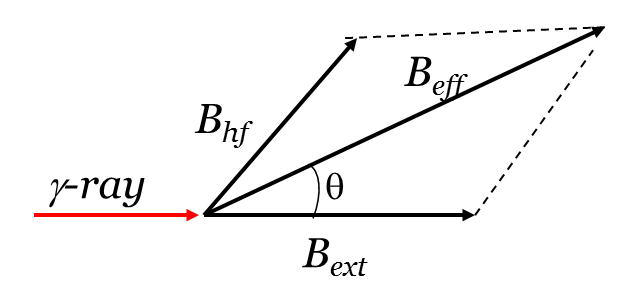


Figure S4. The setup for ^57^Fe Mössbauer spectroscopy and the relation between the hyperfine field (B_hf_) the measured effective nucleus field (B_eff_) and the external field (B_ext_), with evidenced the average canting angle ϑ.

For a thin sample, where thickness effects are negligible and where the direction of the hyperfine field is at random with respect to the gamma-ray direction, the relative area of the Zeeman sextet is in the ratio:

$$3:2p:1:1:2p:3 (5)$$

where *p* depends on the eventual canting angle *ϑ*:

$$p=\frac{2\sin^{2} \theta}{1+\cos^{2} \theta} (6)$$

By normalizing the total area to 1, the area of lines 2-5 (A_2,5_) is equal to:

$$A_{2,5}=\frac{1}{2}\sin^{2} \theta(7)$$

In case of the absence of canting effect, lines 2-5 disappear. On the other hand, from their residual intensity, the corresponding average canting angle ϑ over the entire particle is determined [5]:

$$\theta=arcsin\sqrt{2A_{2,5}} (8)$$

Table S1. From the fitting of Mössbauer spectra the mean isomer shift (δ), the mean quadrupole shift (2ε) the mean effective field (B_eff_) the average canting angle (θ_cant_), the mean hyperfine field (B_hf_), and the % area of each component are evaluated.

| Sample | Site | δ (mm s^-1^) | 2ε (mm s^-1^) | B_eff_ (T) | θ (°) ± 10° | B_hf_ (T) | % |
| --- | --- | --- | --- | --- | --- | --- | --- |
| MAG1 | A | 0.41 | -0.08 | 56.8 | 36 | 50.5 | 39 |
|  | B | 0.49 | -0.01 | 44.8 | 40 | 51.2 | 61 |
| MAG2 | A | 0.36 | -0.03 | 60.1 | 19 | 52.6 | 39 |
|  | B | 0.49 | -0.03 | 45.3 | 19 | 52.9 | 61 |
| MAG3 | A | 0.32 | 0.00 | 60.5 | 17 | 52.9 | 38 |
|  | B | 0.48 | 0.00 | 46.2 | 25 | 53.6 | 62 |
| MAG4 | A | 0.33 | -0.04 | 60.0 | 27 | 53.0 | 38 |
|  | B | 0.49 | -0.05 | 46.0 | 28 | 53.2 | 62 |

## Spin-canted shell thickness

Coey’s model [6] describes magnetic nanoparticles with an inner core region with perfect collinear spins surrounded by an outer shell of canted spins. For particles with diameter *D*, the shell of canted spin, with thickness *t*, can be considered responsible for the whole canting angle ϑ extracted from Mössbauer data.

Normalizing all the spectra to 1, the area of 2,5 lines is:

$$A_{2,5}=\frac{1}{2}\sin^{2} \vartheta=\frac{q}{3} (9)$$

Where *q* is the fraction of spin canted and it is equal to:

$$q=1-\left( 1-\frac{2t}{D} \right)^{3} (10)$$

Hence, the thickness *t* of the canted shell can be calculated as [5]:

$$t=\left[ 1-\left( 1-\frac{3}{2}\sin^{2} \vartheta\right)^{\frac{1}{3}} \right]\frac{D}{2} (11)$$

# MAG3 ZFC and FC M(H) curves

The exchange coupling between a magnetically disordered frozen surface and the inner ordered core of a small nanoparticle often gives rise to an exchange bias effect [7], i.e. a shift of the M(H) curve measured after a field cooling process. This effect is visible in our sample MAG1 as reported in **Fig. 6** in the main manuscript. Levy et al. [8] noted an exchange bias effect in 8 nm particles prepared by seed-mediated growth similarly to MAG3. Their sample exhibited regular crystalline structure when analyzed by high-resolution TEM. Nevertheless, the structure exhibited internal strain due to inhomogeneous growth of the shell initiated from multiple nucleation sites. This was responsible for the local canting and disordered magnetic structure, eventually producing the exchange bias effect. We have tested this behavior in sample MAG3, recording an M(H) curve at 5 K after a field cooling of 1 T. However, no sign of bias has been observed in our case (**Fig. S5**).





Figure S5. M(H) curves measured at 5 K for sample MAG3 after a zero field cooling (black dots and line) and a field cooling with 1 T (red triangles and line) from room temperature.

Levy et al. used a reduced temperature for the growth of the shell (250°C vs 280°C for the particle core) aiming to induce a slow and controlled growth of the shell layer. In our case, the sample MAG3 shell was grown at a higher temperature (300 °C). Possibly this process allowed for a better organization of the atomic structure of the shell, preventing the residual internal strain and allowing a homogenous growth of the whole particle.

# References

1. Aslibeiki B, Kameli P, Ehsani MH, et al (2016) Solvothermal synthesis of MnFe2O4 nanoparticles: The role of polymer coating on morphology and magnetic properties. Journal of Magnetism and Magnetic Materials 399:236–244. https://doi.org/10.1016/j.jmmm.2015.09.081

2. Wohlfarth EP (1958) Relations between Different Modes of Acquisition of the Remanent Magnetization of Ferromagnetic Particles. Journal of Applied Physics 29:595–596. https://doi.org/10.1063/1.1723232

3. O’Grady K, Chantrell RW (1992) Remanence Curves of Fine particles Systems I: Experimental Studies

4. Peddis D, Cannas C, Musinu A, et al (2013) Beyond the Effect of Particle Size: Influence of CoFe 2 O 4 Nanoparticle Arrangements on Magnetic Properties. Chemistry of Materials 25:2005–2013. https://doi.org/10.1021/cm303352r

5. Tronc E, Prené P, Jolivet JP, et al (1998) Spin Canting in γ-Fe2O3 Nanoparticles. Hyperfine Interactions 112:97–100. https://doi.org/10.1023/A:1011092712136

6. Coey JMD (1971) Noncollinear Spin Arrangement in Ultrafine Ferrimagnetic Crystallites. Physical Review Letters 27:1140–1142. https://doi.org/10.1103/PhysRevLett.27.1140

7. De Toro JA, Vasilakaki M, Lee SS, et al (2017) Remanence Plots as a Probe of Spin Disorder in Magnetic Nanoparticles. Chemistry of Materials 29:8258–8268. https://doi.org/10.1021/acs.chemmater.7b02522

8. Levy M, Quarta A, Espinosa A, et al (2011) Correlating Magneto-Structural Properties to Hyperthermia Performance of Highly Monodisperse Iron Oxide Nanoparticles Prepared by a Seeded-Growth Route. Chemistry of Materials 23:4170–4180. https://doi.org/10.1021/cm201078f
